# Getting the right helper opens a new avenue for NLR engineering

**DOI:** 10.1093/plcell/koad201

**Published:** 2023-07-20

**Authors:** Leiyun Yang

**Affiliations:** Assistant Features Editor, The Plant Cell, American Society of Plant Biologists, Rockville, MD, USA; Department of Plant Pathology, College of Plant Protection, Key Laboratory of Integrated Management of Crop Diseases and Pests, Ministry of Education, Nanjing Agricultural University, Nanjing 210095, China; The Key Laboratory of Plant Immunity, Nanjing Agricultural University, Nanjing 210095, China

Crop pathogens are a persistent threat to global food security. One of the most sustainable and effective ways to mitigate crop disease is through breeding disease-resistant crops using resistance genes, which often encode nucleotide-binding leucine-rich repeat (NLR) proteins. Plant NLRs recognize pathogen virulence proteins called effectors that initiate downstream immune responses, including programmed cell death for preventing pathogen spread or growth. NLRs can work as pairs, with one acting as a sensor for perception of effectors and the other as a helper for transducing immune signaling (Adachi et al. 2019). This is exemplified by the rice NLR pair Pik-1/Pik-2 that recognizes AVR-Pik, an effector from the fungal pathogen *Magnaporthe oryzae* and that have evolved multiple allelic variants, including Pikp-1/Pikp-2 and Pikm-1/Pikm-2. Some sensor NLRs contain noncanonical integrated domains that serve as effector baits to sense effector proteins. Pik-1 belongs to this category with its integrated heavy metal–associated (HMA) domain that recognizes AVR-Pik. Engineering of integrated domains like HMA can expand effector recognition specificity of the corresponding NLRs (De la Concepcion et al. 2019; Liu et al. 2021). However, in the case of HMA domains, changes can result in auto activity or constitutive cell death due to the incompatible pairing of sensors and helpers (Maidment et al. 2023), which presents a bottleneck to develop new disease resistant crops.

In this issue, **Adam R. Bentham, Juan Carlos De la Concepcion, and colleagues (Bentham et al. 2023)** reveal the mechanistic basis for the incompatibility between Pik-1 and Pik-2 variants and demonstrate the potential of Pikp-2 as a facilitator for engineering new effector recognition specificities.

The authors previously found that mismatching sensor Pikp-1 and helper Pikm-2 results in constitutive cell death without an effector binding to the integrated HMA domain of Pikp-1 (De la Concepcion et al. 2021). To examine the contribution of the HMA domain to NLR incompatibility, they deleted the HMA domain (replacing it with an unrelated integrated domain) to create the Pikp-1^ΔHMA^ variant. Like Pikp-1, Pikp-1^ΔHMA^ coexpressed with Pikm-2 exhibited constitutive cell death, whereas no cell death was observed when coexpressing with Pikp-2 (see Figure). They further swapped the HMA domains of Pikm-1 and Pikp-1 to create the chimera sensors Pikm-1^pHMA^ and Pikp-1^mHMA^. Coexpressing Pikm-2 with Pikm-1^pHMA^ caused constitutive cell death that was abolished when coexpressing with Pikp-1^mHMA^, suggesting that HMA domain contributes to compatibility of the Pik-1/Pik-2 pair. Interestingly, coexpression of Pikp-1^mHMA^ with Pikp-2 was not auto active, revealing that Pikp-2 tolerates more changes in the HMA domain than Pikm-2 without inducing auto activity (see Fig.).

**Figure.**
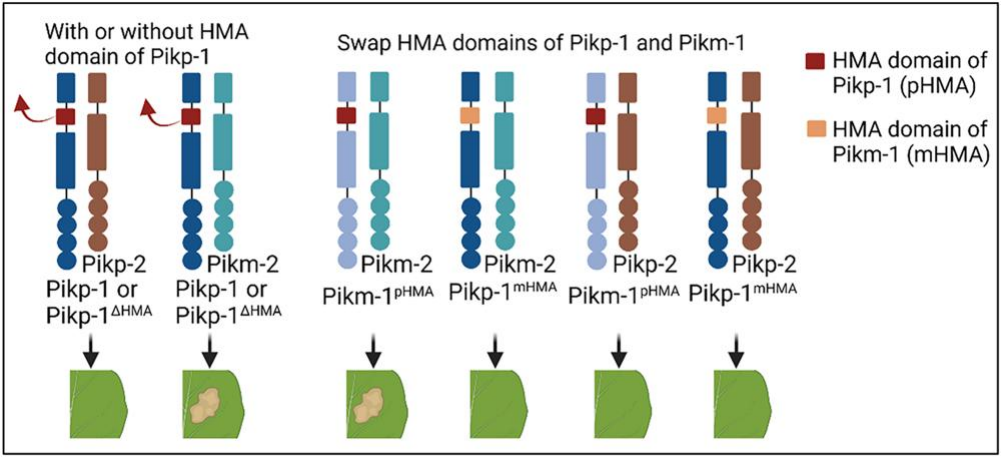
Coexpression of Pikp-1 (with or without the HMA domain) with Pikm-2, but not with Pikp-2, causes constitutive cell death. Swapping the HMA domains of Pikp-1 and Pikm-1 demonstrates that Pikp-2 tolerates more changes in the HMA domain than Pikm-2 without inducing constitutive cell death. The figure was created with Biorender by L. Yang.

These results prompted the authors to test whether Pikp-2 allows for integration of an HMA domain that would normally result in auto activity. They replaced the HMA domain of Pikm-1 with that from the unrelated sensor RGA5 and termed the chimeric protein Pikm-1^RGA5^. The Pikm-1^RGA5^ chimera induced a strong constitutive cell death when coexpressing with Pikm-2, whereas no cell death was observed when coexpressing with Pikp-2. Guided by structural analyses, the authors engineered an AVR-Pik binding (APB) interface in the RGA5 HMA domain responsible for AVR-Pia binding. Co-immunoprecipitation and multicycle or single-cycle kinetics (measured by Surface Plasmon Resonance) assays revealed high binding affinity of 3 AVR-Pik variants and weak binding affinity of AVR-Pia for the APB mutant. Consistently, Pikm-1^APB^ coexpressed with Pikp-2 triggered cell death when challenged with AVR-Pik variants, whereas only a weak cell death response was observed when challenged with AVR-Pia. These data demonstrated that the HMA domain of Pikm-1 is amenable to engineering for expanding effector recognition specificities when pairing with Pikp-2.

Finally, the authors tested the utility of this Pik system in wider aspects of NLR engineering. It was recently shown that a Pik-1-nanobody (single-domain antibody fragment) integrated with nanobodies with affinity for fluorescence proteins can recognize fluorescence proteins when coexpressed with Pik-2 (Kourelis et al. 2023), which opens a new avenue for NLR engineering. However, some of the Pikm-1-nanobody coexpressed with Pikm-2 causes constitutive cell death. The authors found that Pikp-2 significantly alleviated cell death compared with Pikm-2 when coexpressing with Pikm-1-nanobody, supporting the conclusion that Pikp-2 can facilitate NLR engineering.

In summary, this study reveals a new strategy for NLR engineering and advances our understanding of immune signaling. Integration of new effector recognition motifs tailored to specific effector proteins into the Pik system provides a promising approach for engineering precise and efficient disease resistance. In future work, it will be important to test whether this system can provide the desired increase in resistance against *M. oryzae* in rice.
